# Intermittent fetal heart rate monitoring using a fetoscope or hand held Doppler in rural Tanzania: a randomized controlled trial

**DOI:** 10.1186/s12884-018-1746-9

**Published:** 2018-05-04

**Authors:** Paschal Francis Mdoe, Hege L. Ersdal, Estomih R. Mduma, Jeffrey M. Perlman, Robert Moshiro, Peter T. Wangwe, Hussein Kidanto

**Affiliations:** 1 Haydom Lutheran Hospital, Mbulu, Tanzania; 2 Department of Health Science, University of Stavanger, Stavanger, Norway; 3 Department of Anesthesiology and Intensive Care, Stavanger University Hospital, Stavanger, Norway; 4 Department of Pediatrics, Weill Cornell, New York, USA; 5 Department of Research, Stavanger University Hospital, Stavanger, Norway; 6 Muhimbili University of Health and Allied Sciences, Dar es Salaam, Tanzania; 7 Muhimbili National Hospital, Dar es Salaam, Tanzania; 8 Obstetrics and Gynaecology, Haydom Lutheran Hospital, PO box 9000, Haydom, Mbulu Tanzania

**Keywords:** Intermittent fetal heart rate monitoring, Pinard fetoscope, Doppler

## Abstract

**Background:**

Neonatal mortality is a global challenge, with an estimated 1.3 million intrapartum stillbirths in 2015. The majority of these were found in low resource settings with limited options to intrapartum fetal heart monitoring devices. This trial compared frequency of abnormal fetal heart rate (FHR) detection and adverse perinatal outcomes (i.e. fresh stillbirths, 24-h neonatal deaths, admission to neonatal care unit) among women intermittently assessed by Doppler or fetoscope in a rural low-resource setting.

**Methods:**

This was an open-label randomized controlled trial conducted at Haydom Lutheran Hospital from March 2013 through August 2015. Inclusion criteria were; women in labor, singleton, cephalic presentation, normal FHR on admission (120–160 beats/minute), and cervical dilatation ≤7 cm. Verbal consent was obtained.

**Results:**

A total of 2684 women were recruited, 1309 in the Doppler and 1375 in the fetoscope arms, respectively. Abnormal FHR was detected in 55 (4.2%) vs 42 (3.1%). (RR = 1.38; 95%CI: 0.93, 2.04) in the Doppler and fetoscope arms, respectively. Bag mask ventilation was performed in 80 (6.1%) vs 82 (6.0%). (RR = 1.03; 95%CI: 0.76, 1.38) of neonates, and adverse perinatal outcome was comparable 32(2.4%) vs 35(2.5%). (RR = 0.9; 95%CI: 0.59, 1.54), in the Doppler and fetoscope arms, respectively.

**Conclusion:**

This trial failed to demonstrate a statistically significant difference in the detection of abnormal FHR between intermittently used Doppler and fetoscope and adverse perinatal outcomes. However, FHR measurements were not performed as often as recommended by international guidelines. Conducting a randomized controlled study in rural settings with limited resources is associated with major challenges.

**Trial registration:**

This clinical trial was registered on April 2013 with registration number NCT01869582.

## Background

Globally, an estimated 1.2 million fresh stillbirths (FSB) and 2.1 early neonatal deaths (END) occurred in 2013, 98% in low and middle-income countries [1–4]. Currently, the proportion of neonatal deaths constitute 44% of under-five child mortality [5]. Intrapartum hypoxia (often defined as birth asphyxia) has been reported to be associated with as much as 70% of FSB and 60% of END [6–9]. Identification and timely management of intrapartum hypoxia is therefore a priority area for reducing perinatal mortality in low resource countries.

Intrapartum fetal heart rate (FHR) monitoring aims at recognizing the hypoxic fetus as a result of fetal interruption of placental blood flow during labor. FHR tracings can reveal specific information regarding fetal oxygenation. Thus, an abnormal FHR tracing (i.e. late deceleration, severe variability, bradycardia and tachycardia) during the first stage of labor has been associated with fetal hypoxia and fetal acidosis [10–12], and intermittently detected abnormal FHR has been associated with adverse perinatal outcome in a rural setting [13]. Several studies from resource replete settings have reported lower rate of operative deliveries (i.e. cesarean section, vacuum or forceps extraction) when comparing intermittent to continuous FHR monitoring, respectively [14, 15]. Importantly, in these studies, intermittent FHR assessments were performed according to recommended guidelines i.e. optimal frequency and length of measurements.

In low resource settings, FHR monitoring is mainly performed intermittently, using a Pinard fetoscope. However, in 1994 in an urban hospital in Harare, a hand-held Doppler ultrasound was found to be more effective in detecting abnormal FHR and improved perinatal outcome in low risk deliveries as compared to the Pinard fetoscope [16]. The hand-held Doppler allows the care provider to assess FHR quickly, to communicate with the mother who can hear the heart sounds of her baby hence increase maternal confidence concerning the wellbeing of her unborn baby, and it is preferred by women over the use of fetoscope [17, 18]. Main barriers toward the more widely use of Doppler in low-resource countries have been costs and lack of reliable electricity supply. Therefore, a wind-up hand-held Doppler device has been developed using a hand-crank to generate power, that last for about 2 h for every 30-s of cracking [19]. This wind-up Doppler was tested in a low resource setting and found to be accurate and acceptable to mothers as well as midwives [19]. A recent randomized controlled study, comparing the Free-Play Doppler to the Pinard fetoscope in a peri-urban setting in Uganda, reported an increased detection rate of abnormal FHR in the Doppler arm (7.6%) as compared to the Pinard arm (4.7%), but no improvement of perinatal outcome [20].

To our knowledge, intermittent FHR monitoring using Doppler or fetoscope has never been compared in a rural sub-Saharan setting that differs substantially from (peri-) urban settings. The aim of this study was to compare the Free-Play Doppler with the Pinard fetoscope for intermittent FHR monitoring, detection of abnormal FHR and impact on perinatal outcomes in a low-resource rural hospital.

## Methods

We conducted a randomized control trial comparing the Pinard fetoscope and Free-Play Doppler (Free Play, Power-free Education Technology, Pet.og.za) for intermittent FHR monitoring from March 2013 through August 2015. Data collection was carried out at Haydom Lutheran Hospital (HLH), a rural referral hospital in Northern Tanzania. The hospital provides comprehensive emergency obstetric care and basic newborn care to a population of approximately 500,000 people, while the greater reference area covers about 2 million people [21]. Midwives largely conduct uncomplicated deliveries, and doctors on call for 24 h perform operative deliveries. Traditionally, FHR is routinely monitored by the attending midwife using a Pinard fetoscope. For this study, the wind-up hand-held Free-Play Doppler was introduced. All midwives and doctors were initially trained and then retrained after every six months on how to use the Doppler and simultaneously reminded about abnormal FHR patterns and its obstetrical management. Midwives were allowed to use Doppler, if available, also for deliveries not included in the study.

### Study population and randomization

The inclusion criteria are listed in Table 1. Recruitment, information about the study, and verbal consent was obtained by the admitting midwife in the admission room. Women who consented were randomly allocated using sealed opaque envelops. The allocation label was stapled to the patient file, and the selected device (Doppler or fetoscope) used for that woman.

**Table 1:** Inclusion criteria

• In labor (i.e. cervical dilatation ≥3 cm with uterine contractions)	
• Singleton	
• Cephalic presentation	
• Gestation age ≥ 36 weeks	
• Cervical dilatation ≤7 cm	
• Normal FHR at admission	
• Able to give verbal consent	

### Data collection

Research assistants were continuously present in the labor ward, observing every delivery. They worked in three shifts over 24 h. Three research assistants covered each shift; two research assistants were always in the delivery rooms or in the operating theatre; one in the adjacent neonatal area. Altogether 14 research assistants were trained to observe health care workers’ performance related to deliveries and newborns. Findings were recorded on a data collection form. This included labor progress information and FHR categorized as normal (120 to 160 beats/minute), abnormal (< 120 or > 160 beats/minutes), absent, or not measured; specific device used for FHR assessment; neonatal characteristics and interventions in the delivery room, and perinatal outcome (normal, admitted neonatal area, death within 24 h and FSB). The frequency of FHR monitoring was not recorded, though midwives were expected to follow WHO guidelines of monitoring FHR every 30 min during the first and after five to 15 min during the second stage of labor. Therefore, categorization of FHR was based on multiple measurements throughout labor. Abnormal FHR was reached after at least one confirmed measurement at any point during labor.

Maternal age was based on self-report of the mother or close relative. Maternal gravidity was the number of pregnancies the woman had ever had including the current one. Gestational age (GA) was based on self-report of the last menstrual period and distance from symphysis pubis to fundus (on admission). Normal term GA at HLH is routinely defined as ≥ 36 weeks (Thus, preterm was defined as GA < 36 weeks and post term as > 42 weeks.

Normal perinatal outcome was defined as a live birth and included all of the following; Apgar score more than seven at one and five minutes, not admitted to the neonatal unit. Intrapartum death/FSB was defined as an Apgar score of zero at both one and five minutes with intact skin and suspected death during labor/delivery i.e. with a FHR heard on admission. Antepartum death/macerated stillbirth (MSB) was defined as an Apgar score of zero at both one and five minutes with macerated skin and suspected death before start of labor. “FHR abnormalities” included abnormal or not detected. There were some cases where the FHR was not measured. “Adverse outcome” included FSB, neonatal deaths within the first 24 h and infants still admitted in the neonatal unit after 24 h.

### Quality assurance of the data

The research assistants were trained, supervised, and continuously re-trained by the first author. All data collection forms were controlled for quality issues, before entering to the computer, including missing information and potential errors, on a daily basis. Data were double entered using SPSS Version 22 by two different data clerks.

### Sample size and statistical consideration

Primary outcome measure was detection of an abnormal FHR. A previous study at HLH revealed an abnormal FHR detection rate of 2.7% in the total cohort when using the Pinard fetoscope [13]. Based on this study, an 80% increase in detection rate was estimated for the Free Play Doppler arm i.e. 4.8%, equivalent to the proportion of abnormal FHR detection when using Pinard in the peri-urban setting in Uganda [20]. An estimated 1277 cases were required in each arm to achieve a power of 80% using a two-sided test with significance level 0.05. A total of 2684 cases were included, 5% more than the calculated sample, to compensate for potentially missing variables. Data was analyzed using the Statistical Package for the Social Sciences (SPSS) v.22. Chi-square calculations and Fisher’s exact test were used to compare categorical variables, and independent samples t-test were used for continuous data.

### Ethical considerations

Ethical clearances were granted by the Muhimbili University of Health and Allied Sciences ethics and publication committee, and the Regional Committee for Medical and Health Research Ethics, Western Norway (2013/110/REK vest). Verbal consent was approved to be used in this trial. Permission to collect data was granted by the management at HLH. The trial was registered in ClinicalTrials.gov Identifier NCT01869582.

## Results

### General characteristics of the whole cohort

**Fig. 1:**
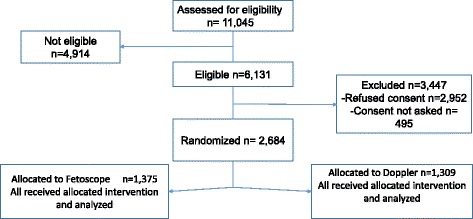
Consort flow diagram

During the study period 11,045 women delivered in HLH (Fig. 1). Mean maternal age was 25.1 ± 6.5 years. Almost 99% had attended antenatal care at least once, and 1.0% reported some type of pregnancy problems. The proportion of preterm delivery was 3.3%. Mean birth weight (BW) was 3202 ± 529 g, and 880 (8%) neonates had BW less than 2500 g. On admission, 114 (1.0%) had an abnormal FHR, 217 (2.0%) had an undetectable FHR, and 1027 (9.3%) were not assessed. During labor, irrespective of device used, abnormal FHR was detected in 387 (3.5%) women. Overall, the mean time interval from admission to detection of an abnormal FHR was 6.5 ± 6.7 h, from the first detected abnormal FHR to birth 120 ± 210 min, and from the final recorded FHR assessment to birth was 92 ± 210 min. The majority of women delivered vaginally, i.e. 8455 (76.6%), whereas 2438 (22.1%) were delivered via caesarean section (CS). Apgar scores less than seven at one and five minutes were encountered in 392 (3.5%) and 66 (0.6%) neonates, respectively. There were150 (1.4%) MSB, 155 (1.4%) FSB, 90 (0.8%) deaths within 24 h after birth and 357 (3.2%) neonates admitted to the neonatal unit for more than 24 h.

A total of 4914 women did not meet the inclusion criteria, 20.9% (1027/4914) of these due to lack of FHR assessment on admission. A sub-population of 6131 women met the inclusion criteria for the study. Of these 3447 (56.2%) women were not included because either they refused (*n* = 2952) or consent was not taken (*n* = 495) (Fig. 1). This resulted in 2684 (43.8%) women who were randomized. A comparison of general characteristics of the women and neonates in the consented and non-consented groups is presented in Table 2. There was a significantly higher incidence of the FHR not being measured during labor in the non-consented group (1.7%) as compared to the consented (0.5%) group, and the CS and FSB rates were higher in the non-consented as compared to the consented group (Table 3).

**Table 2:** Characteristics of women and neonates included in the consented and not consented

Characteristics		Consented *n* = 2684	Not consented *n* = 3447	*P*-value
Maternal Age (Years)	Mean ± SD	25.8 ± 5.7	26.4 ± 6.6	
Maternal Gravida	1	1286 (47.9)	1603 (46.5)	0.274
	2 to 5	975 (36.3)	1276 (37.0)	0.577
	> 5	423 (15.8)	568 (16.5)	0.449
Gestational Age (Weeks)	Mean ± SD	38.3 ± 1.5	37.8 ± 1.4	
	> 40	95 (3.5)	101 (2.9)	0.179
Gender	Male	1471 (56.0)	1824 (53.6)	0.141
	Female	1213 (44.0)	1623 (46.4)	
Birth Weight (Grams)	Mean ± SD	3356 ± 463	33,804 ± 455	
	< 2500	108 (4.0)	180 (5.2)	0.028
	≥ 2500	2576 (96.0)	3267 (94.8)	

Values given are n (%) unless otherwise stated

**Table 3:** Comparison of fetal heart rate (FHR), newborn characteristics and perinatal outcome between the eligible women who consented to participate and those with no consent (refused or not asked for consent)

Characteristics	Eligible population		*p*-value
	Consented *n* = 2684	Not consented *n* = 3447	
FHR abnormalities	112 (4.2)	177 (5.1)	0.078
Abnormal FHR	98 (3.6)	119 (3.5)	0.676
Not measured	14 (0.5)	58 (1.7)	0.001
Time interval from admission to first abnormal FHR assessment (minutes)	466 ± 349	394 ± 357	0.173
Time interval abnormal FHR to birth (minutes)	93 ± 127	104 ± 179	0.624
Time interval last recorded FHR to birth (minutes)	79 ± 187	89 ± 194	0.059
Cesarean section	559 (20.8)	848 (24.6)	0.001
Apgar score < 7 at			
1 Minute	89 (3.3)	113 (3.3)	0.937
5 Minutes	21 (0.8)	33 (1.0)	0.466
Bag mask ventilation	162 (6.0)	239 (6.9)	0.158
Adverse neonatal outcome	67 (2.5)	111 (3.2)	0.094
Fresh stillbirths	7 (0.3)	23 (0.7)	0.023
Early neonatal deaths	14 (0.5)	17 (0.5)	0.890
Admitted neonatal area	46 (1.7)	71 (2.1)	0.471

Values given are n (%)

A total of 1375 and 1309 women were randomized to the fetoscope and the Doppler arm, respectively (Table 4). Maternal mean age was comparable in the two arms. There were more primigravid women in the fetoscope arm as compared to the Doppler arm, 48.5% vs. 45.2%, respectively (*p* = 0.04).

**Table 4:** Characteristics of women and neonates included in the fetoscope and the Doppler arm

Characteristics		Fetoscope arm *n* = 1375	Doppler arm *n* = 1309
Maternal Age (Years)	Mean ± SD	25.4 ± 6.7	24.8 ± 6.2
Maternal Gravida	1	667 (48.5)	592 (45.2)
	2 to 5	492 (35.8)	487 (37.2)
	> 5	216 (15.7)	230 (17.6)
Gestational Age (Weeks)	Mean ± SD	38.1 ± 1.6	38.2 ± 1.7
	> 40	51 (3.7)	51 (3.9)
Gender	Male	770 (56.0)	701 (53.6)
	Female	605 (44.0)	608 (46.4)
Birth Weight (Grams)	Mean ± SD	3286 ± 473	32,804 ± 433
	< 2500	59 (4.3)	49 (3.7)
	≥ 2500	1316 (95.7)	1260 (96.3)

Values given are n (%) unless otherwise stated

Comparison of outcome measures in the two arms are presented in Table 5. The detection of abnormal FHR was higher in the Doppler arm as compared to the fetoscope arm (*p* = 0.109). The time interval from the final recorded FHR (normal or abnormal) to birth was similar in the two arms, but much longer than recommended. The time interval from the first abnormal FHR measure to birth was comparable between the groups. Apgar scores at one and five minutes, neonates receiving bag mask ventilation, and adverse perinatal outcomes were comparable.

**Table 5:** Outcomes of the Infants in the Fetoscope versus the Doppler Groups

Outcomes	Fetoscope *n* = 1375	Doppler *n* = 1309	Effect measure^a^	*p*-value
FHR abnormalities	49 (3.5)	66 (5.0)	1.42 (0.98, 2.03)	0.064
Abnormal FHR	42 (3.1)	56 (4.2)	1.38 (0.93, 2.04)	0.109
Not measured FHR	7 (0.5)	10 (0.7)	1.5 (0.57, 3.93)	0.40
Time interval admission to first abnormal FHR assessment (minutes)	520 ± 386	429 ± 321	90.2 (−63.9, 244.3)	0.248
Time interval abnormal FHR to birth (minutes)	90.8 ± 122	95.1 ± 132	−4.34 (−58.2, 49.5)	0.873
Time interval last recorded FHR to birth (minutes)	79.1 ± 185	79.5 ± 188	−0.43 (−14.6, 13.8)	0.952
Cesarean Section	286 (20.8)	273 (20.9)	1.00 (0.87, 1.16)	0.972
Bag mask ventilation	82 (6.0)	80 (6.1)	1.03 (0.76, 1.38)	0.872
Apgar Score < 7				
1 Minute	46 (3.3)	43 (3.3)	0.98 (0.65, 1.48)	0.921
5 Minutes	11 (0.8)	10 (0.8)	0.95 (0.40, 2.24)	0.914
Adverse perinatal outcome	35 (2.5)	32 (2.4)	0.96 (0.59, 1.54)	0.867
Fresh stillbirths	4 (0.3)	3 (0.2)	0.78 (0.17, 3.51)	0.527
Early neonatal deaths	5 (0.4)	9 (0.7)	1.89 (0.63, 5.63)	0.244
Admitted neonatal area	26 (1.9)	20 (1.5)	0.81 (0.45, 1.44)	0.475

Values given are n (%) unless otherwise stated

^a^Effect measure = Risk Ratio for categorical variables and Mean Difference for continuous variables

Among the 8361 deliveries not included in the randomization study, the type of device used for FHR assessments was noted in 6360 women. The fetoscope was preferred in 4104 (64.5%) cases and Doppler in 1036 (12.4%) cases. No device was used in 1220 (19.2%) cases.

## Discussion

This trial failed to demonstrate significant differences in detection of FHR abnormalities and 24-h perinatal outcomes between Pinard fetoscope versus the Free-Play Doppler when used for intermittent FHR assessments during labor. However, several other important findings related to intermittent FHR monitoring and major challenges associated with conducting a randomized trial in such a rural setting are highlighted.

Doppler was almost 1.4 times more likely to detect abnormal FHR as compared to the fetoscope, however, this was not statistically significant, representing a potential type 2 error. Furthermore, abnormal FHR detection rates, in both arms, were lower in this study as compared to previous studies of similar design [16, 20]. Mahomed et al. reported 32% abnormal FHRs and decreased perinatal mortality when using a standard Doppler device as compared to Pinard (which had 15% abnormal FHRs) [16]. This study was conducted at the National hospital in Harare, Zimbabwe, at a time with fairly good health services (1994), and appointed research midwives were engaged in the study, increasing the ability to follow the recommended frequency for FHR measurements [16]. In 2012–13, Byaruhanga R et al. compared the same devices as used in our study in a peri-urban setting [20]. FHR abnormalities were detected in 7.6% vs 4.7% of the deliveries in the Free Play Doppler and Pinard arm, respectively, but with no differences in perinatal outcome. They speculate that there might have been an undocumented delay between recognition of fetal distress and obstetrical actions. Our study was conducted in a rural setting, and intermittent FHR assessments were done by the midwives.

In addition, several other important factors may have influenced our results. Notably this was the initial time that a randomized trial was conducted in this rural hospital, with unique factors that limited the recruitment and consent seeking process. Thus in this setting women typically arrive late in labor, and many low-risk women were not eligible due to late arrival with cervical dilatation ≥7 on admission. In addition, FHR assessment was not performed in 9% of admissions, which led to the exclusion of these women. Therefore, only 55% of the total cohort fulfilled the inclusion criteria. In addition, only 44% of these eligible women agreed to participate in the study, creating a potential selection bias. There might be several reasons behind the low consent rates. First, women in labor may not be capable of receiving information and making informed decisions. Second, the midwife/patient ratio in this hospital is low, leading to a considerable time constraint for midwives on duty, which may have influenced their ability to spend adequate time recruiting women (8% of the eligible women were never approached for consent). [22, 23] Third, some midwives may have had strong preferences for which device they trusted the most, and therefore wanted to use, potentially affecting their willingness to recruit participants, and to follow the study protocol. Fourth, laboring women might have felt dependent on the midwife at the time of obtaining consent, and this may have influenced their free choice.

Comparing the eligible non-randomized with the randomized group, several important differences stand out. First, more non randomized women did not have FHR assessed during labor, second, time from detection of an abnormal FHR to birth was longer, and third there was a higher incidence of CS (24.6%) and FSB (0.7%) in the non-randomized group. These differences may represent a positive Hawthorne effect of being included in the study. The qualities of FHR assessments followed by timely obstetrical interventions seem to be enhanced in the trial population, with either device used, as compared to daily routine care. Interestingly, CS rate in the non-eligible sub-population, when including high-risk deliveries, was almost identical to the CS rate in the trial population, i.e. 20.9% vs 20.8%, respectively. Of the women in the not-eligible group, 2% were admitted with an absent FHR, FHR assessments were not performed in as many as 21% of admissions, time intervals from admission to birth were shorter (many of these women arrived late in labor), but time intervals from detection of an abnormal FHR to birth were considerably longer. Adverse outcomes were highest in this not-eligible group, maybe reflecting less timely expedited deliveries.

There are several limitations to this randomized trial, reflecting a typical rural sub-Saharan African setting. We report a potential selection bias and a potential positive Hawthorn effect of being enrolled in the trial. Furthermore, a 1:1 midwife/patient ratio was impossible to achieve; therefore, FHR measurements were likely not performed according to recommended guidelines, based on the fact that more than 9% of the women never had a FHR assessed, and that the mean time interval from the last FHR assessment to birth was approximately 1.5 h, much longer than recommended, a finding consistent with previous reports [22, 23]. Complying with the recommended guidelines for optimal intermittent FHR monitoring is reported to require a 1:1 midwife/patient ratio [24–26], and our findings are not representative of hospitals where FHR measurements are done according to guidelines for intermittent FHR assessments. Finally, at HLH, fetoscopes (often homemade) have been common equipment for FHR assessments for decades. Although many hand-held wind-up Dopplers were provided, available also for women not enrolled in the trial, Doppler was rarely used outside the trial. In addition, the fetoscope was used to verify FHR in the Doppler arm more often than the opposite, i.e. Doppler used to verify FHR in the fetoscope arm. This may be explained by an uncertainty related to the new device that also may have influenced the results.

The strength of the study is firstly that it represents a rural hospital with all the typical challenges related to clinical care and patient population, as in other rural low-resource settings. Secondly, at the same time, a unique research infrastructure has been established in parallel to the clinical operations, employing separate staff, not involved with clinical management. Trained research assistants have continuously observed and collected data from every birth since 2009, standard operating procedures regulate all research operations, rigorous data quality control systems involve several levels and different people (manually and electronically), and double independent entry of all data is performed locally, before external control. This comprehensive research set-up has enabled collection of high quality data obtained from all deliveries, including patients not involved in the randomized trial. Finally, a randomized trial design provides high level quality evidence.

## Conclusion

This randomized trial fails to demonstrate significant differences in detection of abnormal FHRs and perinatal outcomes, comparing a fetoscope with a hand-held Doppler for intermittent FHR assessment, in a rural sub-Saharan hospital, with a low midwife/patient ratio. FHR measurements were not performed as often as recommended in the international guidelines, and we speculate that the true frequency of FHR abnormalities is higher in both arms. Conducting a randomized trial in a rural low resource setting is associated with major challenges and requires a minimum setup as described above.

## Abbreviations

BW: Birth weight
CS: Cesarean section
CTG: Cardiotocograph
ENND: Early neonatal death
FHR: Fetal heart rate
FSB: Fresh stillbirth
GA: Gestation age
HLH: Haydom Lutheran Hospital
MSB: Macerated stillbirth
NIMR: National institute of medical research
REK-vest: Regional committee for medical and health research ethics western Norway
SPSS: Statistical package for the social sciences
WHO: World health organization
